# The relationship between the neutrophil-to-lymphocyte ratio and diabetic retinopathy in adults from the United States: results from the National Health and nutrition examination survey

**DOI:** 10.1186/s12886-022-02571-z

**Published:** 2022-08-17

**Authors:** Xiaojie He, Shanshan Qi, Xi Zhang, Jiandong Pan

**Affiliations:** School of Ophthalmology and Optometry, Eye Hospital, Wenzhou Medical University, Wenzhou, Zhejiang China

**Keywords:** Neutrophil-to-lymphocyte ratio, Diabetic retinopathy, Diabetes mellitus, NHANES

## Abstract

**Background:**

Diabetic retinopathy (DR) is a common complication of diabetes mellitus (DM). Systemic inflammation is intimately associated with DR. The neutrophil-to-lymphocyte ratio (NLR) index is a relatively new indicator of inflammation.

**Methods:**

This cross-sectional study was carried out among adults with DM based on the National Health and Nutrition Examination Survey (NHANES) from 2009 to 2016. NLR was presented as absolute neutrophil counts/ absolute lymphocyte counts. The relationship of NLR levels to DR was analyzed using multivariable logistic regression.

**Results:**

There were 2772 eligible subjects extracted from the NHANES. In the multivariate analysis, NLR was related to the risk of DR after adjustment for potential confounders. The association between NLR levels and DR was nonlinear, with an inflection point of 4.778. Compared with the baseline values, NLR was not statistically significant on the right side of the inflection point (1.000, 0.914 to 1.094, 0.9974) but was positively associated with DR on the left side (1.236, 1.132 to 1.349, < 0.0001).

**Conclusions:**

NLR reflects systemic inflammation that may increase the risk of DR. NLR positively correlates with DR when its value is less than 4.778.

## Introduction

Diabetic retinopathy (DR) is a microvascular complication of diabetes mellitus (DM) and is a primary cause of acquired blindness among working-age individuals [1]. Various mechanisms and factors mediate DR development, including pregnancy, diabetic nephropathy, obesity, family history, blood glucose fluctuations, hyperlipidemia, chronic diabetes, hypertension, and hyperglycemia [2–4]. The pathogenesis of DR is not fully understood; however, several studies suggested that inflammation plays an important role [5–7]. Many epidemiological studies highlighted the association between chronic inflammation and DM [8, 9]. Chronic inflammation may contribute to the development of microangiopathy and macroangiopathy in patients with diabetes [10].

Several lines of evidence suggest that routine blood tests might provide adequate information to perform risk stratification [11, 12]. Specifically, peripheral blood leukocytes such as lymphocytes, neutrophils, basophils, eosinophils, and monocytes all have unique biological functions in systemic inflammation [13]. The neutrophil-to-lymphocyte ratio (NLR) can indicate systemic inflammation [14]. NLR represents the ratio of neutrophils to lymphocytes in peripheral blood, which integrates different but complementary immune pathways in circulating blood. Increased NLR may be the result of increased neutrophils, which can adhere to endothelial cells, resulting in vascular endothelial damage and widespread chronic inflammation [12, 15]. Furthermore, lymphocytes are the main cells of the body’s immune response and have the ability to regulate inflammatory responses [16].

NLR has been used for mortality stratification in major cardiac events [17, 18], indicating infectious and inflammatory status or postoperative complications [19, 20]. NLR was also considered a prognostic and predictive factor for DM and its complications [21, 22]. Wang et al. found that NLR was related to DR in patients with diabetes who had no associated family history in China [23]. Nevertheless, further research is required to determine whether this is the same in different regions, populations, and types of diabetes. Therefore, our study further evaluated the relationship between NLR and DR through a larger sample data from the United States (US).

## Methods

### Sources of the data and samples

The National Health and Nutrition Examination Survey (NHANES) is a cross-sectional, stratified, multistage probability cluster survey conducted in the US and the details of NHANES have been reported elsewhere [24]. The data for this study was derived from the NHANES between 2009 and 2016, including potential risk factors and nutrition in the civilian, non-institutionalized, American population. The National Center for Health Statistics Institutional Review Board (NCHS IRB/ERB) approved the protocol (NCHS ERB protocols #2011–17). All participants provided informed consent. After they were interviewed at home, the participants underwent health examinations in mobile examination centers. Subjects who were aged less than 18 years, pregnant or lactating, were excluded. The physiological and medical statuses of the participants were evaluated and required laboratory tests were completed. Four cycles of NHANES surveys were used to evaluate the relationship between DR and NLR. All adult patients (≥18 y) with type 1 and type 2 DM were included. The exclusion criteria were: (a) missing neutrophil or lymphocyte data, (b) incomplete Patient 90 Health Questionnaire-9 (PHQ-9) [25], and (c) the use of nonsteroidal anti-inflammatories or corticosteroids.

### Variables

Using automated hematology analyzing devices, neutrophil and lymphocyte counts were measured and expressed as × 10^3^ cells/μl. NLR was expressed as absolute neutrophil count/lymphocyte counts. The selected indicators included age, gender, marital status, systolic blood pressure (SBP), waist circumference, triglycerides (TC), body mass index (BMI), high-density lipoprotein (HDL), diastolic blood pressure (DBP), low-density lipoprotein (LDL), C-reactive protein (CRP), glycosylated hemoglobin A1c (HbA1c), hemoglobin, neutrophil count, lymphocyte count, NLR, the ratio of family income to poverty (PIR), stroke, coronary heart disease (CHD), and heart failure (HF).

### Statistical analyses

The χ2 test and independent-sample t-test were used to compare the differences in features at baseline in the non-DR and DR groups for categorical and continuous variables, respectively. Multivariate logistic regression analysis was conducted to evaluate the relationship between DR and NLR. Odds ratio (OR) and 95% confidence intervals (CI) were calculated. Based on the STROBE statement [26], the fully-adjusted, minimally adjusted, and unadjusted analyses were demonstrated. The matched odds ratio was changed by more than 10% when the covariances were added to this model [27]. Non-linear relationships were identified using generalized additive models (GAM). If the correlation was non-linear, a two-piecewise linear regression model was established based on the smoothing plot. When the ratio of DR and NLR fell on a smooth curve, automatic calculation of the inflection point was achieved using the recursive method, in which the maximum model likelihood estimation was applied [28]. Subgroup analysis of the associations between NLR and DR was carried out by applying the stratified linear regression model. *P* < 0.05 (two-sided) was considered statistically significant. R language (http://www.R-project.org) along with EmpowerStats software (http://www.empowerstats.com/en/, X&Y solutions, Inc., Boston, MA) were employed for all the analyses.

## Results

### Characteristics of the subjects

A total of 2772 patients had DM and met the inclusion criteria, including 1348 females and 1424 males. The average age was 61.3 ± 13.2 years. There were 637 (23.0%) patients suffering from DR. Characteristics at baseline are displayed in Table 1. Participants with DR had elevated CRP, HbA1c, neutrophil counts, NLR, diabetes duration, and lower lymphocyte counts. Male sex, stroke history, CHD, and HF were more common in DR patients (*p* < 0.05). Age, years, SBP, DBP, BMI, waist circumference, TC, HDL, LDL, triglycerides, hemoglobin, PIR, and marital status showed no significant differences between diabetic patients with DR and those without.

**Table 1:** Characteristics of participants in the NHANES (2009—2016) by diabetic retinopathy

Characteristics	Diabetes Mellitus (*n* = 2135)	Diabetic Retinopathy (*n* = 637)	*P* value
Age, years	61.1 ± 13.3	61.9 ± 13.0	0.199
SBP, mmHg	131.6 ± 19.6	132.1 ± 20.5	0.642
DBP, mmHg	68.5 ± 14.2	66.9 ± 14.3	0.075
BMI, kg/m^2^	32.3 ± 7.4	32.9 ± 8.2	0.098
Waist circumference, cm	109.1 ± 15.6	110.9 ± 17.8	0.081
TC, mg/dl	180.8 ± 45.2	177.0 ± 45.0	0.063
HDL, mg/dl	48.4 ± 14.4	47.5 ± 14.9	0.198
LDL, mg/dl	101.8 ± 35.9	97.7 ± 34.5	0.085
Triglycerides, mg/dl	148.7 ± 110.8	149.3 ± 116.4	0.940
CRP, mg/dl	0.5 ± 0.7	0.6 ± 0.8	0.047*
HbA1c, %	7.4 ± 1.8	7.9 ± 1.9	< 0.001**
Hemoglobin, g/dl	13.6 ± 1.6	13.4 ± 1.6	0.544
Neutrophil count, 10^9^ /l	4.6 ± 1.7	4.8 ± 1.8	0.018*
Lymphocyte count, 10^9^ /l	2.2 ± 0.9	2.1 ± 0.8	0.002**
NLR	2.4 ± 1.5	2.7 ± 1.7	< 0.001**
PIR	2.2 ± 1.5	2.1 ± 1.5	0.202
Diabetes duration, years	10.1 ± 4.9	12.9 ± 3.0	< 0.001**
Gender, n (%)			< 0.001**
Male	1056 (49.5)	368 (57.8)	
Female	1079 (50.5)	269 (42.2)	
Marital Status			0.348
Married, %	1237 (58.2)	372 (58.6)	
Widowed/divorced, %	676 (31.8)	211 (33.2)	
Never married, %	214 (10.1)	52 (8.2)	
Stroke, n (%)			< 0.001**
Yes	173 (8.1)	80 (12.6)	
No	1954 (91.9)	555 (87.4)	
CHD, n (%)			0.012*
Yes	213 (10.1)	86 (13.7)	
No	1898 (89.9)	544 (86.3)	
HF, n (%)			< 0.001**
Yes	175 (8.3)	92 (14.6)	
No	1945 (91.7)	538 (85.4)	

*SBP* Systolic blood pressure, *DBP* Diastolic blood pressure, *BMI* Body mass index, *TC* Total cholesterol, *HDL* High density lipoprotein, *LDL* Low density lipoprotein, *CRP* C-reactive protein, *HbA1c* Glycosylated hemoglobin A1c, *NLR* Neutrophil to lymphocyte ratio, *PIR* Poverty income ratio, *CHD* Coronary heart disease, *HF* Heart failure

*P < 0.05, **P < 0.01

### NLR was related to the risk of DR

A variety of models were constructed to evaluate the relationship of NLR with DR (Table 2) after potential confounding factors were adjusted. An increased NLR level was related to the elevated risk of DR in the model (OR = 1.112, 95% CI: 1.054–1.173, *P* = 0.0001). After several factors were adjusted (age, gender, BMI, PIR, diabetes duration, marital status, stroke, CHD, and HF), increased NLR levels were shown to be related to the elevated risk of DR (OR = 1.076, 95% CI: 1.015–1.142, *P* = 0.0146). The same trend was observed in sensitivity analysis when NLR was considered a categorical variable (tertiles) (*P* = 0.0116).

**Table 2:** Association between NLR and diabetic retinopathy

Variable	Crude Model		Model I		Model II	
	OR (95%CIs)	*P* value	OR (95%CIs)	*P* value	OR (95%CIs)	*P* value
NLR	1.112 (1.054, 1.173)	0.0001**	1.098 (1.040, 1.159)	0.0007**	1.076 (1.015, 1.142)	0.0146*
NLR (Tertiles)						
< 1.76	1.0(ref)		1.0(ref)		1.0(ref)	
≥ 1.76, < 2.57	1.204 (0.962, 1.507)	0.1047	1.162 (0.927, 1.457)	0.1924	1.111 (0.862, 1.433)	0.4164
≥ 2.57	1.520 (1.222, 1.891)	0.0002**	1.438 (1.152, 1.796)	0.0013**	1.371 (1.066, 1.765)	0.0141*
*P* trend	0.0002**		0.0012**		0.0116*	

*OR* Odds ratio, *CI* Confidence interval

Models were derived from logistic multivariate regression models

Crude model adjust for: none

Adjust I model adjust for: age, gender

Adjust II model adjust for: age, gender, BMI; poverty income ratio; diabetes duration; marital status; stroke; coronary heart disease; heart failure

*P < 0.05, **P < 0.01

### Non-linear relationship analyses

**Fig. 1:**
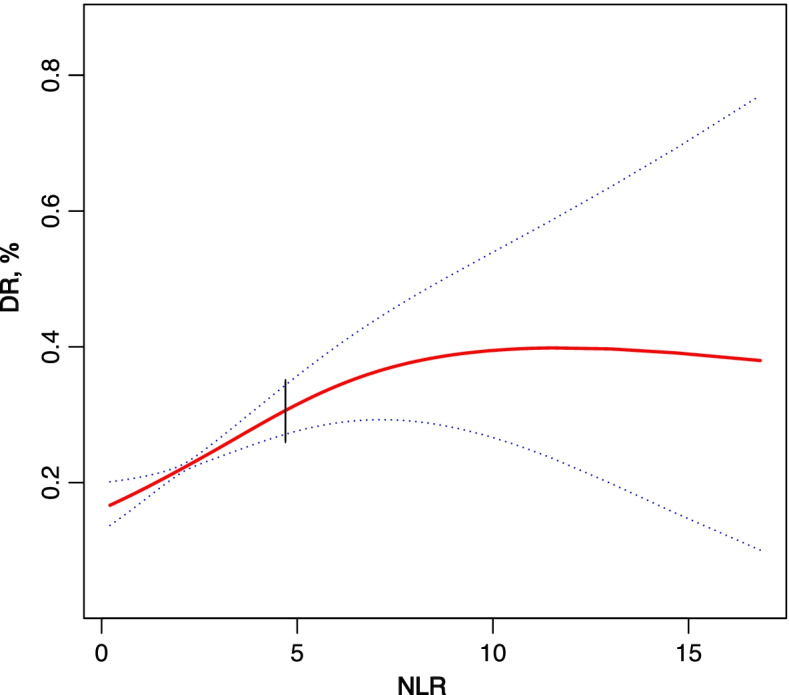
The relationship between NLR and DR. Black vertical lines: inflection point 4.778; Solid line: occurrence probability of DR; Dotted line: 95% confidence interval curve

The non-linear relationship between NLR and DR was also explored (Fig. 1). A two-piecewise linear regression model was used. The inflection point was 4.778. On the right side of the inflection point, *P*-value, 95% CI, and the effect size were 0.9974, 1.000, and 0.914–1.094, respectively. On the left side, a positive association between NLR and DR in the inflection point was found (1.236, 1.132–1.349, < 0.0001), and there was no significant difference in the inflection point on the right side (1.000, 0.914–1.094, 0.9974).

### Subgroup analysis

The results of the subgroup analysis are displayed in Table 3. There was no difference in NLR levels among most pre-specified subgroups in DR participants, except for hemoglobin and waist circumference. A high NLR level had an independent relationship with DR in high waist circumference (≥107.6 cm), and low hemoglobin (< 13.6 g/dl), and the corresponding ORs (95% CIs) were 1.459 (1.054, 2.020) and 1.699 (1.252, 2.304), respectively.

**Table 3:** Subgroup analysis of the associations between NLR and diabetic retinopathy

	NLR			*P* for interaction
	< 1.76	≥1.76, < 2.57	≥2.57	
Gender				0.2452
Male	1.0(ref)	1.259 (0.922, 1.719)	1.361 (1.005, 1.843)	
Female	1.0(ref)	1.046 (0.750, 1.458)	1.606 (1.166, 2.213)	
Marital Status				0.1623
Married	1.0(ref)	1.120 (0.828, 1.515)	1.721 (1.291, 2.294)	
Widowed/divorced	1.0(ref)	1.199 (0.820, 1.753)	1.183 (0.809, 1.729)	
Never married	1.0(ref)	1.833 (0.872, 3.854)	1.609 (0.747, 3.462)	
Age, years				0.2286
< 63	1.0(ref)	1.293 (0.946, 1.767)	1.823 (1.332, 2.495)	
≥ 63	1.0(ref)	1.103 (0.798, 1.524)	1.278 (0.941, 1.737)	
SBP, mmHg				0.4644
< 130	1.0(ref)	1.138 (0.811, 1.596)	1.593 (1.145, 2.216)	
≥ 130	1.0(ref)	1.229 (0.887, 1.702)	1.225 (0.891, 1.683)	
DBP, mmHg				0.7890
< 68	1.0(ref)	1.269 (0.888, 1.812)	1.411 (1.001, 1.988)	
≥ 68	1.0(ref)	1.091 (0.797, 1.493)	1.332 (0.975, 1.819)	
BMI, kg/m^2^				0.1015
< 31.2	1.0(ref)	1.019 (0.735, 1.413)	1.520 (1.112, 2.078)	
≥ 31.2	1.0(ref)	1.308 (0.952, 1.796)	1.364 (0.996, 1.867)	
Waist circumference, cm				0.0241*
< 107.6	1.0(ref)	1.010 (0.726, 1.406)	1.365 (0.982, 1.899)	
≥ 107.6	1.0(ref)	1.386 (0.991, 1.938)	1.459 (1.054, 2.020)	
Hemoglobin, g/dl				0.0085**
< 13.6	1.0(ref)	1.217 (0.881, 1.679)	1.699 (1.252, 2.304)	
≥ 13.6	1.0(ref)	1.198 (0.876, 1.639)	1.352 (0.988, 1.849)	
PIR				0.2918
< 1.7	1.0(ref)	1.326 (0.958, 1.835)	1.588 (1.152, 2.190)	
≥ 1.7	1.0(ref)	1.120 (0.791, 1.586)	1.540 (1.105, 2.145)	
TC, mg/dl				0.8835
< 174	1.0(ref)	1.235 (0.897, 1.699)	1.325 (0.970, 1.809)	
≥ 174	1.0(ref)	1.112 (0.802, 1.541)	1.629 (1.189, 2.232)	
HDL, mg/dl				0.3069
< 46	1.0(ref)	1.302 (0.946, 1.793)	1.478 (1.077, 2.027)	
≥ 46	1.0(ref)	1.082 (0.781, 1.497)	1.492 (1.095, 2.031)	
LDL, mg/dl				0.9532
< 96	1.0(ref)	1.148 (0.700, 1.883)	1.285 (0.804, 2.053)	
≥ 96	1.0(ref)	1.486 (0.908, 2.431) 1	1.595 (0.968, 2.631)	
Triglycerides, mg/dl				0.6060
< 120	1.0(ref)	1.391 (0.852, 2.274)	1.468 (0.919, 2.343)	
≥ 120	1.0(ref)	1.187 (0.737, 1.911)	1.428 (0.889, 2.293)	
HbA1c, %				0.4348
< 7	1.0(ref)	1.265 (0.886, 1.806)	1.403 (0.988, 1.993)	
≥ 7	1.0(ref)	1.147 (0.855, 1.540)	1.605 (1.207, 2.133)	
CHD				0.4167
Yes	1.0(ref)	1.325 (0.683, 2.571)	0.930 (0.486, 1.781)	
No	1.0(ref)	1.149 (0.903, 1.463)	1.621 (1.283, 2.047)	
Stroke				0.6780
Yes	1.0(ref)	1.569 (0.741, 3.320)	2.026 (0.991, 4.141)	
No	1.0(ref)	1.151 (0.908, 1.458)	1.446 (1.148, 1.823)	
HF				0.4004
Yes	1.0(ref)	1.349 (0.651, 2.796)	1.088 (0.552, 2.145)	
No	1.0(ref)	1.148 (0.904, 1.458)	1.513 (1.197, 1.913)	

*NLR* Neutrophil to lymphocyte ratio, *SBP* Systolic blood pressure, *DBP* Diastolic blood pressure, *BMI* Body mass index, *PIR* Poverty income ratio, *TC* Total cholesterol, *HDL* High density lipoprotein, *LDL* Low density lipoprotein, *HbA1c* Glycosylated hemoglobin A1c, *CHD* Coronary heart disease, *HF* Heart failure

*P < 0.05, **P < 0.01

## Discussion

This study is the first to identify the relationship between DR and NLR levels in the US population to the best of our knowledge. NLR was related to DR, as demonstrated by the adjusted model. The same trend was seen when NLR was set as a categorical variable. Using the regression model and GAM, we found that the association between NLR and DR was non-linear. The correlations were different on different sides of the inflection point (NLR = 4.778). Compared with the NLR at baseline, there was no significant difference in the inflection point on the right side. However, NLR was positively correlated with DR on the left side.

The association of systemic inflammation and progression of DR is a research hotspot. It has been reported that bacterial infections and high levels of lipopolysaccharide activity are associated with increased risk of severe DR in type 1 diabetes [29]. Chronic inflammation is a pivotal factor in initiating and promoting diabetes and may contribute to the development of microangiopathy and macroangiopathy in diabetic patients [10]. Leukocytes and the subgroups in peripheral blood correlated with microvascular and macrovascular complications in patients with diabetes [30]. DR is a common microangiopathic complication in diabetes. Several studies found that inflammation had an essential role in different stages of DR [31, 32]. DR, a microvascular complication commonly seen in diabetes, is characterized by the formation of macular edema and new blood vessels [33] and disturbed crosstalk between glial cells and the loss of neurons [34].

NLR can indicate the status of systemic inflammation according to complete blood count. Neutrophil counts generally increase as the inflammatory disease progresses. However, the count does not increase in some diseases, such as cachexia, which leads to a “false negative” result during the evaluation of disease progression. Lymphocyte counts decrease when inflammatory disease deteriorates. However, a decreased lymphocyte cannot precisely indicate the actual status of the disease because this kind of change is relatively delayed [35, 36]. Recent research has demonstrated that NLR was a more reliable parameter in diagnosing or predicting survival than using lymphocyte or neutrophil count alone [37]. As the disease progresses, NLR increases simultaneously, especially in inflammation-related diseases [38].

Using two factors simultaneously, NLR is considered a new marker for evaluating systemic inflammation [22]. An elevated level of neutrophils may contribute to an increase in NLR, leading to damages in vascular endothelium, and in turn, causing severe chronic inflammation [12, 15]. Lymphocytes work as an essential part of systemic immune responses. A relatively higher level of CD4 T cells was shown to ameliorate atherosclerosis [16]. Thus, NLR may indicate systemic inflammation in patients with DR.

The relationship between DR and blood inflammation indexes has been widely discussed [39–41]; the present study further investigated the relationship, and the application of NLR in patients with DR. Our findings are partially similar to those of Wang et al. [23], but they are also somewhat different. We both showed that NLR was associated with DR; however, there was an inflection point in this index. NLR positively correlated with DR within a specific range as the value increased; beyond this range, there was no correlation. A high NLR indicated that the patient was in the acute phase of the disease with acute changes such as an acute infection [14, 42]. Therefore, it may not have a high correlation with the occurrence or development of DR. Moreover, unlike the former study, ours include a larger sample size.

There were some limitations in our study. First, causality cannot be established in a cross-sectional study; this would require prospective studies. Second, data were obtained from only one blood test. Serial testing provides more information because of the short life of blood cells. Third, the lack of information about DR severity and some other important indicators may have affected our conclusions. However, these limitations may be balanced by our strengths, including a large sample size, which represents the population in the US, the inclusion of ethnic/racial minorities, a wide range of ages, precise data and information of covariates, and a sufficient number of events.

## Conclusion

NLR levels correlated with an elevated risk of DR. NLR positively correlated with DR when its value was less than 4.778. However, these should be further confirmed by conducting prospective studies.

## Data Availability

All data were downloaded from the NHANES website (http://www.cdc.gov/nchs/Nhanes).
